# Deubiquitinases in Neurodegeneration

**DOI:** 10.3390/cells11030556

**Published:** 2022-02-05

**Authors:** Abudu I. Bello, Rituparna Goswami, Shelby L. Brown, Kara Costanzo, Taylor Shores, Shefaa Allan, Revan Odah, Ryan D. Mohan

**Affiliations:** Division of Biological and Biomedical Systems, School of Science and Engineering, University of Missouri—Kansas City, Kansas City, MO 64110, USA; aib6wy@mail.umkc.edu (A.I.B.); rgrvv@mail.umkc.edu (R.G.); slby9b@mail.umkc.edu (S.L.B.); kmc7b9@mail.umkc.edu (K.C.); tast4q@mail.umkc.edu (T.S.); sa7vh@mail.umkc.edu (S.A.); revan.odah@mail.umkc.edu (R.O.)

**Keywords:** deubiquitinase, ubiquitin, ubiquitin-specific protease, neurodegeneration, *Drosophila*

## Abstract

Ubiquitination refers to the conjugation of the ubiquitin protein (a small protein highly conserved among eukaryotes) to itself or to other proteins through differential use of ubiquitin’s seven internal linkage sites or the amino-terminal amino group. By creating different chain lengths, an enormous proteomic diversity may be formed. This creates a signaling system that is central to controlling almost every conceivable protein function, from proteostasis to regulating enzyme function and everything in between. Protein ubiquitination is reversed through the activity of deubiquitinases (DUBs), enzymes that function to deconjugate ubiquitin from itself and protein substrates. DUBs are regulated through several mechanisms, from controlled subcellular localization within cells to developmental and tissue specific expression. Misregulation of DUBs has been implicated in several diseases including cancer and neurodegeneration. Here we present a brief overview of the role of DUBs in neurodegeneration, and as potential therapeutic targets.

## 1. Introduction

Ubiquitin is a small (8.6 kDa) protein that is well conserved among eukaryotes. For example, *Drosophila* and human ubiquitin share 100% sequence identity. Such exact conservation through evolution can be interpreted as evidence of a molecule’s critical mechanistic importance for living organisms. In cells, ubiquitin is used to post-translationally modify proteins [1]. This is initiated by activation of ubiquitin by the ubiquitin activating enzyme E1. In a series of reactions, an E1 enzyme activates the carboxyl terminus of ubiquitin by forming a high-energy bond between the cysteine of its active site and the C-terminal Gly76 of ubiquitin [2]. The activated ubiquitin is then transferred to an E2, a ubiquitin-conjugating enzyme, through transthiolation. Finally, an E3, a ubiquitin-protein ligase, covalently attaches ubiquitin to the ε-amino group of a target lysine of the substrate protein. Substrate specificity is mediated by the action of the E3 directly binding the target protein [3]. The result of this is that through E3, ubiquitin is able to form diverse linkages, using different lysine residues or the primary amine at the ubiquitin amino-terminus—all of which can participate in polyubiquitin chain formation [4,5,6,7,8,9,10,11,12,13].

The resulting diversity of conjugation options has enticed researchers to conceptualize these (poly)ubiquitin linkages as comprising a biological “ubiquitin code” that may be deciphered to understand the fate of each modified protein [14,15]. Conceptually, the ubiquitin code works similarly to letters in an alphabet, combining to create a language that conveys specific information based on which ubiquitin residue has been used to conjugate ubiquitin to the modified protein and the length of the chain. For example, K48-linked polyubiquitin chains often target modified proteins to the proteasome for degradation [16], while K63-linked polyubiquitin chains regulate proteasome-independent signaling pathways including DNA repair, inflammatory signaling, and endocytosis [17]. The broader consequences of ubiquitination have been understood from systematic and laborious investigations into how each ubiquitination event leads to specific cellular control of individual proteins. Specifically, M1-linked chains activate nuclear factor kB (NF-kB) signaling, while K11-linked chains regulate proteasomal degradation and intracellular trafficking of many proteins [18]. Long chains of ubiquitin are not the only form of ubiquitin required for signaling, short chains and even monoubiquitylation contribute to a diverse signaling toolbox that triggers cellular processes ranging from epigenetic regulation to proteasomal degradation [19].

Accordingly, ubiquitination causes an incredible spectrum of changes in cellular protein activity including, but not limited to, changes in protein conformation, localization, interactions, and other follow-on post-translational modifications. Ubiquitination also affects chromatin structure and modulates cellular signaling pathways [20,21,22]. Broad generalizations regarding the effects of ubiquitination can be formed by examining the characteristics of linkages at various lysine residues, which can be used to predict the consequences of ubiquitination on a given protein; however, a detailed investigation must be conducted to discern the consequences of ubiquitination on individual proteins. Importantly, the effects of ubiquitination can be interrupted or reversed through the removal of these tags by deubiquitinases (DUBs). This process also produces free ubiquitin molecules, which can then be conjugated to other substrates.

## 2. What Are Deubiquitinases (DUBs)?

DUBs are enzymes that recognize and hydrolyze the bonds linking ubiquitin to substrate proteins or other ubiquitin molecules. The number of DUBs expressed by an organism correlate approximately with its regulatory complexity. For example, 42 proteins are included under the category “deubiquitinases” in the *Drosophila* genetics database “FlyBase” [23,24,25,26,27,28,29,30,31], while the human genome encodes approximately 100 DUBs [32]. DUB activities may be regulated in several ways such as differential expression during development and/or between tissues, restricted subcellular location, or post-translational modification [11,33,34,35,36,37].

DUBs are multi-domain proteins with several structural characteristics. The yeast genome encodes 22 putative DUBs that are subdivided into classes based on their sequence and structural similarity. Five of the seven classes of DUBs in human are conserved in yeast: ubiquitin specific protease (USPs), ovarian tumor-like proteases (OTUs), ubiquitin carboxy-terminal hydrolases (UCHs), Machado–MIU-Containing Novel DUB Family (MINDY), and JAB1/MPN/MOV34 metalloenzymes (JAMM/MPN±) [32]. Below, we briefly discuss the structural features of the DUBs families that are conserved in yeast. The UCH structure includes a catalytic domain with 230 amino acid residues and a papain-like catalytic triad structure [38]. On the other hand, in the second family of DUB, USPs structure includes the core catalytic domain and extensions that are used for substrate or target recognition [39]. The third family of DUBs are the OTUs, specialized for their homology to the ovarian tumor gene in *Drosophila melanogaster*. The OTU structure has a catalytic domain with five β-strands between conserved helical structures. The DUBs of the OTU family are extremely specific for which ubiquitin chains they use as substrate [40]. The fourth family of DUBs is the most recently found—the MINDY family, which stands out due to their distinct catalytic domain structure. Study of the human MINDY DUB structure revealed the catalytic domain of MINDY1 is wrapped around a highly structural hydrophobic pocket [41]. The last family of DUBs is the conserved metalloproteases JAMM which includes a Clu-X-[N]-His-X-His-X[10]-Asp core motif that coordinates the binding of two Zn^2+^ ions [39,42].

The human DUBs are more extensive and can be grouped into seven families based on the homologies of their catalytic domains, with the two DUBs unique to humans being Machado-Joseph domain-containing proteases (MJDs), and the Zinc Finger with UFM1-Specific Peptidase Domain Protein/C6orf113/ZUP1 (ZUP1/ZUFSP) [32,43,44]. The MJD class of DUB share a common cysteine protease domain of ∼180 amino acids, known as the Josephin domain [45,46]. Ataxin-3 is the best characterize protein of the Josephin domain-containing proteins and contains a single Josephin domain at their N terminus plus a flexible C-terminal domain of comparable length [47]. Ataxin-3 enzyme preferentially cleaves long poly-ubiquitin chains over short substrates and the full-length protein preferentially cleaves Lys-63-linked and mixed-linkage ubiquitin chains [48,49]. In addition, ataxin-3 also cleaves adducts at the C terminus of NEDD8, a protein that is closely related to ubiquitin in both structure and sequence [50]. Furthermore, the ZUP1/ZUFSP family of DUB contains a highly conserved motif interacting with ubiquitin (MIU) motif in its central portion [43]. The modular domain organization of ZUFSP contains four *N*-terminal zinc finger (ZnF) motifs and a *C*-terminal peptidase domain with similarity to the UFM1-specific proteases UFSP1/2. ZUFSP has DUB activity by virtue of a *C*-terminal cysteine protease domain distinct from any known DUBs. ZUFSP selectively interacts with and cleaves long K63-linked poly-Ub chains in a manner dependent on its Ub-binding domains, whereas it displays poor activity toward mono- or di-Ub substrates.

The six cysteine DUBs contain members with ubiquitin peptidase activities necessary for the processing and cleavage of linear ubiquitin chains (M1-linked) [51]. The catalytic domains of these cysteine proteases contain ubiquitin-deconjugating isopeptidases that are required for specific substrate binding and linkage formation. In addition, analogous peptidases and isopeptidases have been identified, such as those regulating the ubiquitin-like modifiers (UbLs) SUMO and NEDD8 [51,52]. Non-cysteine JAMM DUBs act primarily in larger complexes, such as the proteasome. There, the JAMM protease Rpn11 recycles ubiquitin from proteins targeted for degradation [51]. In addition, several proteins encoded by bacteria and viruses have confirmed deubiquitinase activity [53].

DUBs play two key roles in the regulation of ubiquitination [32]: removing ubiquitin from substrates and processing ubiquitin’s inactive precursor. Misfolded proteins are a hallmark of many neurodegenerative diseases, underscoring the importance of normal proteostasis as a regulatory mechanism in neural survival and the central nervous system functionality [54,55,56,57,58]. The ubiquitin-proteasome system (UPS) is one of the major protein degradation pathways where abnormal UPS function has been observed in cancer and neurological diseases such as Alzheimer’s disease (AD), Parkinson’s disease (PD), and Huntington’s disease (HD). Intracellular ubiquitin-positive inclusions formed by aggregate-prone neurotoxic proteins are a common feature of many neurodegenerative diseases. This suggests that UPS dysfunction contributes to the accumulation of neurotoxic proteins in neurodegenerative diseases and possibly disease instigation.

## 3. What Are Some Cellular Functions of DUBs?

**Figure 1:**
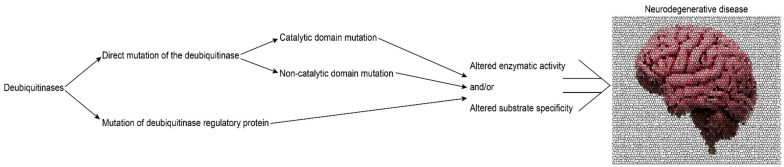
Mechanism of DUB involvement in neurodegeneration. This figure shows three mechanisms of dysfunction in DUBs: (1) direct mutations in the gene encoding the DUB; (2) a central role for the DUB in a ubiquitin-dependent quality control pathway implicated in disease; and (3) involvement of the DUB in handling a specific substrate that is critical to neurodegenerative disorders.

DUBs generate free ubiquitin, trim polyubiquitin chains, and remove ubiquitin from proteins. Through these activities, DUBs act as chief regulators of protein homeostasis. Similar to E3 ubiquitin ligases, DUBs are highly regulated and participate in several cellular functions: gene expression [59]; cell cycle control [60]; chromatin structural dynamics [61]; kinase activation [62]; protein localization; protein trafficking; and protein stability [57]. Furthermore, DUB dysregulation has been linked to diseases, including cancer and neurological disorders [63,64]. The involvement of DUBs in neurological diseases may happen by a three-step process: (1) direct mutations in the gene encoding the DUB; (2) a central role for the DUB in a ubiquitin-dependent quality control pathway implicated in disease; and (3) involvement of the DUB in handling a specific substrate that is critical to a neurodegenerative disease process (Figure 1). Direct mutation in the gene encoding the DUB occurs in Spinocerebellar ataxia type 3 (SCA3) due to CAG-trinucleotide expansion of the gene encoding ATXN3, resulting in polyglutamine expansion of the ATXN3 gene product, which is a deubiquitinase [47]. In other cases, the mutation may be in a gene that encodes a protein that regulates a DUB. An example of this is Spinocerebellar ataxia type 7 (SCA7) which results from CAG-trinucleotide expansion of the ATXN7 gene, leading to polyglutamine expansion of the ATXN7 protein [65]. ATXN7 anchors the DUB USP22 to SAGA, controlling its entry and exit from the complex [33,66]. SCA7 is a progressive neurodegenerative disease characterized by degeneration of the spinocerebellar tract and the retinas [66,67]. The mechanisms of SCA7 and SCA3 are still being deciphered, but in these and in other neurodegenerative diseases it appears that DUB misfunction leads to a series of complex downstream events which can derail important ubiquitin-dependent quality control such as macroautophagy—the intracellular degradation of proteins and organelles [68,69]. The ubiquitin-proteasome system (UPS) controls the levels of proteins regulating autophagy through ubiquitination and degradation, which is essential for autophagic flux [70].

A long-standing mystery for most DUBs is what the specific targets of each DUB are and how the action of the DUB on each substrate is controlled. Some elements controlling substrate selection include linkage specificity [71], target specificity through the catalytic domain [72,73,74], and by the target recognition via ubiquitin-binding domains [63,75,76,77]. While progress has been made over the last decade in understanding their roles and interactors, additional research is needed to uncover the substrates and complete physiological functions of most DUBs (Table 1).

## 4. DUBs in Macroautophagy

Several neurodegenerative diseases have been linked to defects in macroautophagy (the intracellular degradation of proteins and organelles) [68,69]. Many regulators, including the key macroautophagic factor microtubule-associated protein 1A/1B-light chain 3 (LC3), also regulate protein folding and protein–protein interactions. Ubiquitin-like modifier activating enzyme 6 (UBA6) negatively regulates autophagy in coordination with the ubiquitin ligase baculoviral IAP repeat containing 6 (BIRC6) by marking LC3 for polyubiquitination and proteasomal degradation [117]. However, the DUBs responsible for opposing this and stabilizing LC3 have not been fully elucidated [117].

The early stages of macroautophagy machinery formation require establishment of a cytoplasmic double membrane-bound phagophore, a process which is regulated by mammalian target of rapamycin (mTOR). DEP-domain containing mTOR interacting protein (DEPTOR) negatively regulates mTOR by inhibiting its kinase activity. In its normal physiological state, DEPTOR is continuously degraded by the UPS. It is, however, stabilized during nutrient deprivation by the DUB OTUB1, through a ubiquitin aldehyde binding. This leads to mTOR inactivation and assembly of the unc-51-like autophagy activating kinase 1 (ULK1) complex, which is required for phagophore formation [69]. Importantly, ULK1 is positively regulated by USP20 via deubiquitination and stabilization. Beclin 1 (BECN1) is an important regulator of autophagy that is necessary for phagophore membrane expansion and is deubiquitinated and stabilized by USP10 and USP13 [118]. USP33 also positively regulates BECN1 by deubiquitination and promotes its interaction with RAS like proto-oncogene B (RALB), which it also positively regulated, enabling the growth of the phagophore membrane. Consequently, USP14 negatively regulates BECN1 by cleaving its K63-linked polyubiquitin chains instead of its K48-linked chains, marking it for proteasomal degradation [118]. In addition, USP36 plays a key role in the deubiquitination of monoubiquitinated histone 2B (H2B), a mark important for transcriptionally active chromatin, which promotes expression of BECN1 and another important autophagy regulator, autophagy related 14 (ATG14) [119].

Importantly, DUB activity is also required for selective macroautophagy. E3 ligase PARK2-mediated macroautophagy (the selective degradation of damaged mitochondria via macroautophagy) is an example of this, and it is negatively regulated via USP30-catalyzed deubiquitination of mitochondrial outer membrane proteins [120,121]. Ubiquitin-specific protease 8 (USP8) facilitates macro mitophagy by deubiquitinating and stabilizing PARK2 [69]. In yeast, deubiquitination of 60S ribosome subunits by mRNA-binding ubiquitin-specific protease UBP3 induces their selective autophagic [122]. Additional DUBs with key roles in macroautophagy have been identified through their interactions with other proteins, such as USP8 (discussed above), which was characterized after its identification as a PARK2 interactor [69].

Together, these examples indicate that lysosome-dependent, self-degradative pathways are widely controlled by deubiquitination and that several diverse DUBs regulate these autophagic processes. In addition to their effects on macroautophagy, several individual DUBs have been identified that may play important mechanistic roles in neurodegenerative diseases, and therefore, represent potential therapeutic targets to alleviate these conditions.

## 5. DUBs Are Involved in Several Neurodegenerative Diseases

**Figure 2:**
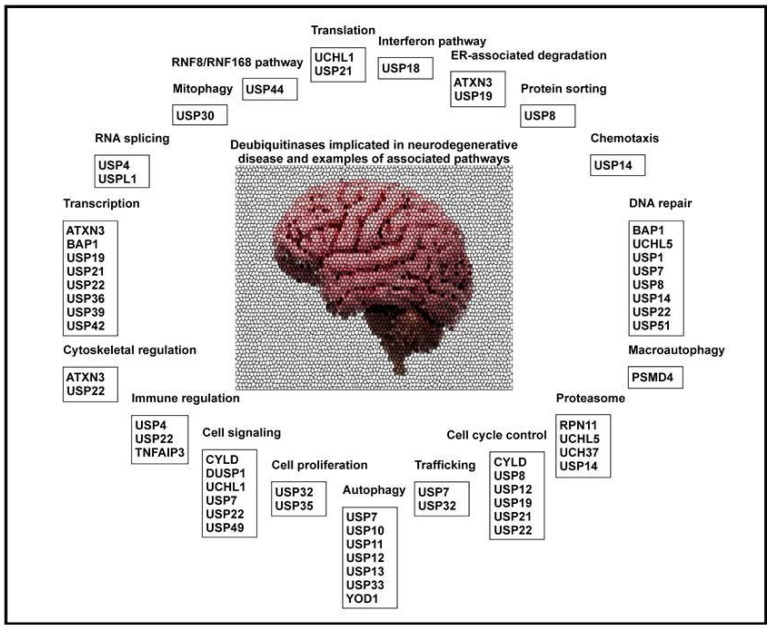
Deubiquitinases implicated in neurodegenerative diseases and associated pathways.

In neurons, mitophagy can be initiated by the depolarization of damaged mitochondria, leading to PARK2 recruitment. Accumulation of PARK2 inhibits both the damaged mitochondrion from fusing with healthy mitochondria and tags them for degradation by autophagosomes. Misregulation of this pathway is linked to axonal degeneration that is characteristic of Parkinson’s disease, which arises from accumulation of dysfunctional mitochondria in the axons [123,124]. DUBs contribute to mitophagy signaling as well (Figure 2). DUBs are typically nuclear or cytoplasmic; however, co-staining with a known mitochondrial protein revealed mitochondrial associations for USP30 and USP35. Cells depleted of USP30 and USP35 exhibit higher levels of mitochondria in lysosomes following depolarization, indicating increased PARK2-mediated mitophagy [124]. This suggests that USP30 and USP35 act to inhibit mitophagy.

### 5.1. DUBs in Neuropathy

Misregulation of DUBs can contribute to neuropathy which in turn leads to neurodegeneration [102,125]. For example, the clinical symptoms of the SCA3 include progressive ataxia due to cerebellar and brainstem degeneration [126]. A common feature observed in SCA3 patients is peripheral neuropathy and axonopathy [126]. In patients with either SCA3 or SCA7, axonopathy comes after neuropathy due to the degeneration of anterior horn neurons and dorsal root ganglia neurons. Though peripheral neuropathy is a problem in ataxia patients, it is present along with other clinical symptoms which get progressively worse over time and are often diagnosed with nerve conduction studies and needle electromyography [127]. Evidence from nerve conduction studies can be a useful biological marker for the progression of the disease [127].

### 5.2. USP6

Aberrant chromosomal translocation of USP6, which encodes a hominoid-specific DUB, is associated with intellectual disability and Asperger’s syndrome [128]. USP6 is a hominoid-specific protein DUB that contains Tre-2/USP6, BUB2, and Cdc16 (TBC) and USP domains, and the USP6 gene is found specifically in humans and orangutans [129]. USP6 TBC and USP domains are highly homologous to TBC1D3 and USP32, respectively [129]. TBC1D3 is linked to human intelligence through its enhancement of neural progenitor cell generation and cortical folding during development [130]. Moreover, USP6 increased synaptic function and N-methyl-D-aspartate receptor (NMDAR) expression when ectopically expressed in mouse brain. Consistently, USP6 depletion in human embryonic stem cell-derived neurons was associated with reduced NMDAR expression and function, suggesting that USP6 promotes cognitive enhancement [131].

These findings and others suggest that DUBs are more directly involved in neurodegeneration than currently acknowledged, underscoring the importance of characterizing their functions, interactors, substrates, and localization.

### 5.3. USP7

Protein homeostasis is essential for proper cellular function, and its perturbation results in proteotoxicity, which is implicated in the pathogenesis of several neurodegenerative diseases including amyotrophic lateral sclerosis (ALS) [132]. To counter the effects of proteotoxicity, organisms have evolved elaborate quality-control mechanisms to remove misfolded and aggregated proteins [133,134]. USP7 is a suppressor of proteotoxicity [135]. The transforming growth factor (TGF) β-mothers against decapentaplegic homolog (SMAD) pathway is involved in protein quality control and mediates USP7′s effects on the clearance of misfolded proteins [135]. USP7 deubiquitinates NEDD4 like E3 ubiquitin protein ligase, which ubiquitinates SMAD2, and influences the SMAD-mediated transcriptional activity that promotes protein quality control [136]. Furthermore, high SMAD2 levels are found in the tissues of patients with ALS, suggesting that defects in this protein quality control pathway are involved in its pathophysiology [135,137]. USP7 also interacts with ATXN1, which is associated with spinocerebellar ataxia type 1 (SCA1), an autosomal dominant neurodegenerative disorder characterized by several neurological defects and symptoms [91].

Furthermore, misregulation of USP7 may be involved in oncogenesis and viral disease [138,139]. USP7 regulates p53, a gene found to be mutated in 50–60 percent of cancers [140,141]. USP7 regulates p53 directly by ubiquitinating it, and indirectly through MDM2 stabilization [142]. MDM2 is an E3 ligase of p53, and USP7 protects MDM2 from proteasomal degradation by stabilizing it through specific binding interactions [143,144,145]. Consequently, in addition to inhibiting p53 transcription, MDM2 ubiquitinates p53 and causes its proteasomal degradation, which results in reduced p53 expression in cancer cells [136,146]. Put together, enhancing USP7–MDM2–p53 interaction may lead to tumor development, and disrupting the interaction could be a future therapeutic target in cancer. Collectively, this information suggests a regulatory role for USP7 in neurodegeneration and cancer.

### 5.4. USP8

Parkinson’s disease (PD) is a progressive neurodegenerative disease characterized by resting tremor, bradykinesia, rigidity, and other symptoms that decrease quality of life. Motor symptoms of PD result from the selective loss of dopaminergic neurons in the substania nigra pars compacta that projects into the dorsal striatum, composed of the caudate and putamen [147,148].

PD is characterized by the formation of inclusions known as Lewy bodies and neuronal death. Misfolding of synuclein alpha (SNCA) is commonly observed, which leads to cognitive dysfunction and the risk of dementia at an early age [149,150,151]. PD SNCA inclusions typically contain K63 ubiquitin chains. However, these chains are decreased in dopaminergic neurons, and the amount of USP8 in their Lewy bodies is inversely related to the extent of K63 ubiquitination [94]. PD SNCA inclusions typically contain K63 ubiquitin chains. However, these chains are decreased in dopaminergic neurons, and the amount of USP8 in their Lewy bodies is inversely related to the extent of K63 ubiquitination [94].

The leptin receptor (LEPR) regulates synapses, neuronal plasticity, cognition, and cortical volume, and has been associated with memory maintenance and depression. USP8 also deubiquitinates LEPR, and USP8 overexpression increases glutamatergic synapse formation in hippocampal areas and the maintenance of brain development in a LEPR-dependent manner [152]. USP8 also has a regulatory function in AD. Misregulation of BACE1 (beta-secretase1) is responsible for the β-amyloid that accumulates in AD, and BACE1 stabilization depends on USP8 [153]. USP8 depletion has been correlated with decreased levels of BACE1 and an increase in its ubiquitination in human neuroglioma cells [96].

### 5.5. USP13

In AD, increased USP13 is observed in the post mortem cortex [154]. USP13 depletion increases SNCA ubiquitination and tau hyperphosphorylation, leading to granule formation. In PD, however, USP13 is increased in the nigrostratium. USP13 regulates PARK2 activity, and its inhibition eliminates the deubiquitination of proteins responsible for aggregate formation in Lewy bodies and tangles [101]. The findings suggest that USP13 could be an effective therapeutic target for neurodegenerative diseases [154].

### 5.6. USP14

USP14 is a proteosome-associated protein involved in cellular quality control [155]. Proteosomes are key proteolytic machinery used by cells to achieve quality control of several intracellular proteins, including aggregation proned ones [156]. Protein aggregation is a hallmark of several diseases, including AD and PD, ALS, dementia with Lewy bodies, frontotemporal dementia, and Huntington’s disease. USP14 alongside UCH37 and RPN11 (proteosome-associated DUBs) mediate the stepwise removal of Ub chain from the distal end of the proteosome [157]. This gradual trimming of the bound Ub is thought to weaken the proteosome/Ub interaction, thereby delaying the degradation in the proteosome. USP14 gene deletion or chemical treatment with USP14 inhibitors was found to result in accelerated degradation of various target substrates [157]. Studies have touted the potential roles of USP14 in the brain. For example, USP14 deletion, characterized by cerebellar ataxia, was found to result in increased GABAA receptor levels in Purkinje cells [158], and considering GABAARs mediate a majority of the fast synaptic inhibition in the mammalian brain, one might reason that USP14 regulation of GABAARs is central to maintaining output neurons of the cerebellar cortex and the regulation of motor coordination in Purkinje cells. Together, these findings suggest that the USP14-mediated regulations may be directly related to various human diseases including neurodegeneration, predicting USP14 as a potential therapeutic target.

### 5.7. USP22

USP22 is associated with the regulation of gene expression, cell growth, the cell cycle, regulation of complexes important for cytoskeleton dynamics, maintenance of cell identity, non-homologous end joining, immune responses, cancer, blindness, diabetes, male infertility, and neurodegenerative diseases [66,159,160,161,162]. It is a component of the Spt-Ada-Gcn5-Acetyltransferase (SAGA) complex, which contains two enzymatic subunits—the GCN5 acetyltransferase and the USP22 deubiquitinase—housed in separate modules. SAGA is best characterized as a chromatin-modifying complex and is a well-known and critical transcriptional coactivator [163]. USP22 contributes to the activation of gene expression by deubiquitinating histones, particularly monoubiquitinated H2B [164]. USP22-initiated deubiquitination of H2B is associated with increased transcription of multiple genes; however, the role of USP22-mediated deubiquitination of H2A is less clear [66,160,161,162].

Ataxin 7 (ATXN7) anchors USP22 to SAGA [65]. CAG triplet expansion in ATXN7 results in a polyglutamine expansion in ATXN7 that causes spinocerebellar ataxia type 7 (SCA7), a progressive neurodegenerative disease characterized by degeneration of the spinocerebellar tract and the retinas [67]. An SCA7 mouse model expressing polyglutamine-expanded ATXN7 displayed impaired USP22 activity, and subsequent effects on the expression of integral target genes that may contribute to SCA7 [165]. Interestingly, in the absence of ATXN7, USP22 is released from SAGA, causing a gain of function effect and consequent decreases in ubiquitinated H2B [162]. In *Drosophila,* ATXN7 loss mimics the phenotypes generated by overexpression of a polyglutamine-expanded amino-terminal truncation of human ATXN7: reduced life span, reduced mobility, and retinal degeneration [166]. These seemingly contradictory observations suggest that a delicate balance of USP22 activity is required, which is maintained by ATXN7.

Although misregulated gene expression is the most obvious explanation for SCA7 phenotypes, a causative set of SAGA-regulated genes has not yet been discovered, leading to a search for non-chromatin substrates of USP22. Recent data collected by our laboratory and others show that USP22 functionally interacts with proteins outside of the SAGA complex [66]. Thus far, interactions between USP22 and non-histone substrates typically result in protein stabilization through increased protection from proteasomal degradation [159]. It is not yet clear if any known USP22 substrates can explain the phenotypes observed upon its mutation.

### 5.8. USP25

Down’s syndrome, caused by trisomy of chromosome 21, is the single most common risk factor for early-onset AD [167], and USP25, which is encoded by a gene on chromosome 21, plays an important role in this disease causation. Recently, researchers developed a mouse model of Down’s Syndrome and AD by crossing 5 × FAD mice (an AD mouse model) with Dp16 mice (a newly-developed mouse model with a trisomy in chromosome 16, which is homologous to human chromosome 21), and observed that trisomy increased neuroinflammation [168]. In this model, increased expression of USP25 resulted in microglial activation, which induced synaptic and cognitive dysfunctions. Prolonged microglial activation induces secretion of proinflammatory cytokines, activating inflammatory signaling cascades and initiating neurotoxic effects during the neurodegeneration process [37,169]. USP25 deletion reduced neuroinflammation and rescued synaptic and cognitive dysfunction by reducing excess cytokine production and consequential inflammation [168]. Endoplasmic reticulum-associated protein degradation (ERAD), which eliminates identified misfolded or abnormal proteins, is counteracted by USP25, which opposes synoviolin 1-catalyzed ubiquitination [170]. Decreased levels of USP25 were observed during ER stress. Together, these changes destabilize amyloid precursor protein in AD. Its rapid degradation leads to β-amyloid formation [171]. These findings suggest the need to maintain a delicate balance of USP25 and its potential role in neurodegeneration [170].

### 5.9. USP46

USP46 is involved in regulation of glutamate receptors, deubiquitinating them at the synapse to prevent their proteasomal degradation [14]. In mammals, USP46 is expressed in the hippocampus, amygdala, cerebellum, and other prefrontal cortex components, and has functions in synaptic plasticity, synaptic transmission, and in neuronal morphogenesis [32]. In addition, USP46 regulates the alpha-amino-3-hydroxy-5-methyl-4-isoxazole-propionic acid receptor (AMPAR) [167]. AMPAR accumulation at synapses is a major molecular mechanism underlying synaptic plasticity, a process associated with performance of higher brain functions such as learning and memory, with its defects leading to neurological disorders and neurodegenerative diseases [172]. Neuronal overexpression of USP46 results in significant reduction in AMPAR ubiquitination, accompanied by reduced rate of AMPAR degradation and an increase in surface AMPAR accumulation. By contrast, knockdown of USP46 by RNAi leads to elevated AMPAR ubiquitination and a reduction in surface AMPARs at synapses in neurons. Consistently, miniature excitatory postsynaptic currents recordings show reduced synaptic strength in neurons expressing USP46-selective RNAi [173]. These results demonstrate USP46-mediated regulation of AMPAR ubiquitination and turnover, may play an important role in synaptic plasticity and brain function.

#### 5.9.1. Ubiquitin Carboxy-Terminal Hydrolases

Ubiquitin carboxy-terminal hydrolase L1 (UCHL1) is extensively involved in neurodegenerative disorders in mice and mammals, and aggregates into inclusion bodies in both AD and PD [86,174,175]. UCHL1 is involved in several neurological functions, and deubiquitinates polyubiquitinated substrates via proteasomal degradation [175,176]. UCHL1 knock-out mice display decreased synaptic vesicles, increased tubulovesicular structures in their axons, and muscle degeneration [177]. In an AD mouse model, reduced monoubiquitination was observed upon loss of UCHL1 [86]. A related DUB, UCHL3, a homolog of UCHL1, is involved in spatial and working memory regulation in mice [178]. In a knockout experiment in mice, it was observed that *Uchl3* mutant mice had significant learning deficiency when compared with the wild type mice [178]. Another member of the UCH subfamily thought to be involved in neurodegenerative disease due to its proteasomal association is UCHL5/UCH37. RPN11, UCH37, and USP14 are the three essential DUBs associated with the 19S regulatory particle of the human proteasome [179,180]. UCH37 removes ubiquitin sequentially from the distal end of the Lys48-linked polyubiquitin chain with the potential to rescue ubiquitinated substrates from proteasomal degradation and was first identified as a component of the 26S proteasome that is thought to be involved in the editing of polyubiquitinated protein substrates [181,182].

#### 5.9.2. ATXN3

ATXN3 is a member of the MJD family of proteins that is evolutionarily conserved. ATXN3 is a small, but ubiquitously expressed protein within the body and is localized widely throughout the cell [183]. The MJDs are a small class of DUBs consisting of four members (ATXN3, ATXN3L, JOSD1, and JOSD2) [131]. The most studied member of this group is ATXN3 [48,184]. ATXN3 is one of the two Josephin proteins primarily found in mammals, and its polyglutamine expansion causes SCA3 disease [47]. ATXN3 is involved in protein quality control pathways, where it helps with proteasomal targeting of substrates by editing their Ub chains [47]. ATXN3 regulates the Ub status of many proteins, and its overexpression in cells is associated with lower levels of highly-ubiquitinated species, a phenomenon requiring ATXN3 catalytic activity [184,185]. Furthermore, RNAi knockdown of ATXN3 leads to increased levels of highly-ubiquitinated species [47,186]. These studies suggest a role for ATXN3 in protein quality control [187]. 

Several possible mechanisms have been proposed to explain the pathogenic role of polyQ-expanded ATXN3, including protein aggregation and transcriptional dysregulation. Genes involved in multiple signal transduction pathways were found to be differentially expressed in ATXN3 null mouse embryonic fibroblasts compared to control [188]. Particularly, the Eph receptor A3 (Efna3), a receptor protein-tyrosine kinase in the Ephrin pathway that is highly expressed in the nervous system, was found to be the most differentially upregulated gene. These findings suggest a role for ATXN3 in the regulation of Ephrin signaling. The study further showed that in the absence of ATXN3, Efna3 upregulation leads to hyperacetylation of histones H3 and H4 at the Efna3 promoter, which is associated with decreased levels of HDAC3 and NCoR in ATXN3 null cells. Collectively, these suggest a normal role for ATXN3 in transcriptional regulation of signaling pathways that may be of relevance to disease processes in SCA3.

## 6. What Are the Mechanisms Underlying Deubiquitinase Misfunction in Neurodegenerative Diseases and What Are the Consequences of Their Misregulation?

DUBs are fundamental for various cellular processes and their activities are closely regulated, and consequently, their dysfunction is linked to malignancies and neurological diseases [189,190]. Neuronal accumulation of insoluble protein aggregates is associated with several neurodegenerative diseases, including PD, AD, dementia, and polyglutamine repeat diseases such as SCA7 [191]. DUB expression and localization in humans is often tissue specific. Furthermore, DUBs can also directly recognize ubiquitin, as seen in some DUBs with auxiliary Ub-binding domains [51]. DUBs regulate ubiquitination signals and play vital roles maintaining neuronal protein homeostasis and signaling [188]. Ubiquitin signals are widely detected in cells including the brain, highlighting the importance of these post-translational modifications in cells and neural tissue [40]. Ubiquitin signals are widely detected in cells including the brain, highlighting the importance of these post-translational modifications in cells and neural tissue [192]. Recent studies in *Drosophila melanogaster* intercepted crosstalk between the SAGA deubiquitinase module (which contains USP22), and the Wiskott-Aldrich syndrome protein family verprolin homolog (WAVE) regulatory complex (WRC) [66]. The WRC promotes actin polymerization, suggesting a correlation between the SAGA deubiquitinase module and cytoskeletal dynamics. This observation may help explain the phenotypes, neurodegeneration, and blindness observed in Drosophila with deubiquitinase module mutations.

Studies of the Drosophila fat facets (*faf*), a homolog of the human USP9X, provided the first in vivo example of a substrate-specific deubiquitination pathway. *Faf* encodes a ubiquitin-binding protein with a clear ortholog in vertebrates [193]. Flies with *faf* mutations display defective eye development, which is linked to increased photoreceptors in their ommatidia due to *faf* loss of function in neighboring non-photoreceptor cells [194]. These mutations have a maternal-effect phenotype, as embryos from homozygous *faf* mothers do not reach the syncytial blastoderm stage. This indicates that the deubiquitinating activity of *faf* is vital to its function in Drosophila since alleles encoding a catalytically inactive form failed to rescue a *faf*-null mutation. Introducing mutant alleles of a 20S proteasome subunit (the α2 subunit) strongly suppressed the *faf* mutant phenotype [195,196], suggesting that *faf* limits ubiquitination and degradation of one or more regulators of eye development.

## 7. Future Directions

Ongoing efforts to develop therapeutics targeting DUBs and proteasomal pathways are promising, emphasizing their critical roles in cellular regulation and signaling. Targeting DUBs is of particular interest because of their cell type and substrate specificities [197]. Specific inhibitors have been identified for many, including USP1, USP7, USP14, USP22, USP30, and UCHL1 [197,198,199,200]. Still, there is much to be learned to fully understand the consequences of pharmacologically modulating DUB function.

Studies have revealed much about the enzymatic properties of DUBs and their role in the nervous system. For most DUBs, however, their precise substrate and physiological role in the nervous system are still unknown. Although methods exist to reveal protein stability or ubiquitination modification changes upon DUB loss, differentiating between direct and indirect effects is challenging and time-consuming. After a DUB substrate is identified, the consequences of its deubiquitination must be investigated. Examining aspects of protein regulation (e.g., level, localization, post-translational modifications, and function) during ubiquitination and deubiquitination can aid in determining the effects of these processes. A further challenge in determining a DUB’s function is the presence of mutations, which can alter their interactions, stability, localization, and catalytic activity. Additionally, fundamental questions remain regarding how various biological pathways are regulated by DUBs in cells and tissues, how they respond to metabolic, hormonal, and circadian changes, and what the consequences of deubiquitination are for the seemingly endless number of biochemical pathways they control. Our collective understanding of DUBs is still in its infancy; however, current evidence reviewed here indicates a fundamental role for the enzymes in neurodegeneration. Further research is needed to expand our knowledge on the role of DUBs in neurodegeneration, and as possible therapeutic targets.

## Figures and Tables

**Table 1:** Potential roles of DUBs in neurodegeneration.

DUB	Role in Neurodegenration	Subcellular Localization	Substrate/Interactors	Ref.
ATXN3	Implicated in several neurodegenerative diseases, including Machado-Joseph Disease	Endoplasmic reticulum	-	[8,78,79]
BAP1	Enriched in neurons and interacts with the Parkinson’s disease (PD)-associated protein synuclein alpha (SNCA)	Nucleus	-	[80]
CYLD	Negatively regulates nuclear factor κB, involved in neuroinflammation	Plasma membrane	-	[81]
PSMD14	Implicated in PD progression	Cytoplasm	Influences mitophagy	[82]
TNFAIP3	Implicated in the onset of neurodegenerative conditions	Plasma membrane	Inhibits NFκB signaling	[83,84]
UCH-L1	Colocalized in protein aggregates and inclusion bodies associated with PD and AD	-	Regulation of monomeric Ub and lipid transfer proteins	[85,86,87]
UCHL-5	Regulates memory and conditions related to memory defects in mice	Cytoplasm	-	[80]
USP1	-	Nucleus	USP1 reverses PCNA ubiquitination	[88]
USP4	Involved in neuroinflammation	Nucleus	Deubiquitinates TRAF6	[89,90]
USP7	Regulates neuronal differentiation by stabilizing REST, and regulates neonatal lethality and hypoplasia	Nucleus	Involved in ataxin 1 transcription	[91,92,93]
USP8	Stabilizes BACE1, which is involved in β-amyloid production in AD-affected brains	-	-	[94,95,96]
USP10	May be involved in the inflammatory microglia phenotype, which is key to brain diseases including neurodegenerative diseases	Cajal bodies	Increases p53 levels in amyloid-stimulated microglia	[97]
USP11	Impinges on autophagy signaling at multiple sites; its inhibition alleviates symptoms of proteotoxicity, a hallmark of neurodegeneration	Nucelus	Interacts and stabilizes the serine/threonine kinase mTOR	[98]
USP12	Dysregulation of USP12 is implicated in Huntington’s disease	-	Regulates neuronal proteostasis and mutant huntingtin	[99]
USP13	Involved in PD, dementia, and neurological disorders, overexpressed in PD-affected brains, stabilizes parkin RBR E3 ubiquitin protein ligase (PARK2) and SNCA	Endoplasmic reticulum	-	[100,101]
USP14	Interacts with neurotransmitter receptors including gamma-aminobutyric acid type A receptors; mutations lead to defects in neuromuscular junction structure and reduced motor performance	Cytoplasm	-	[102,103,104]
USP18	Expression in the white matter of microglia contributes to microglial quiescence	-	Involved in the activation of STAT1 and other interferon genes and regulates interferon signaling	[105]
USP22	Highly expressed in the human brain and associated with many neurological disorders	Nucleus	Maintains viability of brain glioma cells, mutation results in cell cycle arrest and apoptosis	[106,107]
USP30	Implicated in PD progression	Peroxisomes and mitochondria	Antagonizes PARK2 activity by competing for common outer mitochondrial membrane substrates	[108]
USP33	Associated with axonal guidance receptor Robo1		-	[109]
USP36	Implicated in several neurodegenerative diseases, including Machado-Joseph Disease	Endoplasmic reticulum	-	[110]
USPL1	Colocalized in protein aggregates and inclusion bodies associated with PD and Alzheimer’s disease (AD)	Nucleus	Specifically binds SUMO proteins	[111]
USP9X	Involved in lissencephaly, epilepsy, and X-linked intellectual disability	Centriole	-	[112,113,114]
YOD1	Contributes to pathogenesis of neurodegenerative disease by decreasing ubiquitination and degradation of abnormal proteins		Regulates interleukin-1 by binding with TRAF6	[115,116]

## Data Availability

Not applicable.
